# Preparation and characterization of sequilhos-type biscuits added with almond flour of *Acrocomia intumescens*

**DOI:** 10.3389/fnut.2022.1009455

**Published:** 2022-10-12

**Authors:** Maria Michele Alves da Silva, Maria Karine de Sá Barreto Feitosa, Lúcia Emanuele Barros Teixeira Palitot, Henrique Douglas Melo Coutinho, John Eversong Lucena de Vasconcelos, Francisco Antonio Vieira dos Santos, Cícera Gomes Cavalcante de Lisbôa, António Raposo, Hani A. Alfheeaid, Zayed D. Alsharari, Hmidan A. Alturki, Jwaher Haji Alhaji, Erlânio Oliveira de Sousa

**Affiliations:** ^1^Laboratory of Physical-Chemistry and Microbiological Analysis of Food, Faculty of Technology Cariri (FATEC), Juazeiro do Norte, Brazil; ^2^Laboratory of Microbiology and Molecular Biology, Regional University of Cariri (URCA), Crato, Brazil; ^3^Caririense Postgraduate Center (CECAPE) – College of Dentistry, Juazeiro do Norte, Brazil; ^4^Research Center for Biosciences and Health Technologies (CBIOS), Universidade Lusófona de Humanidades e Tecnologias, Campo Grande, Lisboa, Portugal; ^5^Department of Food Science and Human Nutrition, College of Agriculture and Veterinary Medicine, Qassim University, Buraydah, Saudi Arabia; ^6^Department of Clinical Nutrition, Faculty of Applied Medical Sciences, Tabuk University, Tabuk, Saudi Arabia; ^7^General Directorate for Funds and Grants, King Abdulaziz City for Science and Technology, Riyadh, Saudi Arabia; ^8^Department of Health Sciences, College of Applied Studies and Community Service, King Saud University, Riyadh, Saudi Arabia

**Keywords:** flour, macronutrients, physico-chemical analysis, food technology, sustainability

## Abstract

*Acrocomia intumescens* (“macaúba”) is a species that during processing generates a large amount of waste. The use of this residue for the production of flour for incorporation into food products is a way to minimize the cost and nutritionally enrich the final product. This work aimed to develop and analyze, in terms of physico-chemical and microbiological properties, cookies with macaúba almond cake residual flour. Sequilhos formulations were obtained using three different proportions of flour (2, 4 and 6%). The analysis of the flour allowed to find 4.29% of moisture, 1.13% of acidity, 5.33 of pH, 44.46% of carbohydrates, 28.74% of lipids, 20.06% of proteins and 2.45% of ash. In the analysis of the sequilhos formulations, the following values were found: moisture (5.03 to 8.13%), acidity (0.10 to 0.14%), pH (5.52 to 5.93), carbohydrates (67.17 to 73.37%), lipids (18.77 to 31.77%), proteins (0.85 to 1.92%), ash (0.83 to 0.94%) and total energy value (137.57 to 172,50 Kcal/100g). In microbiological analyzes it was highlighted that the sequilhos presented adequate sanitary conditions. The results indicate almond flour as an ingredient to be incorporated in the preparation of sequilhos, which in turn, presented satisfactory physico-chemical properties and microbiological results.

## Introduction

The species *Acrocomia intumescens* (“macaúba”) is found in several regions of Brazil, including Chapada do Araripe, Cariri Cearense, where there is a vast wealth of fruits and native species such as *Caryocar coriaceum* (pequi), *Orbignya speciosa* (babassu), *Mauritia flexuosa* (buriti), among others (1). It has two edible portions: the pulp is consumed fresh or used to make sweets, creams, jellies, ice cream, flour, biscuits, cakes, and breads; the almonds are consumed fresh or in the form of sweets (2).

Macaúba has a socioeconomic interest as a source of raw material for food, medicinal and industrial purposes, being used mainly for the extraction of oil widely used by the cosmetics industry (1). Mechanical extraction by pressing is the way most used by industries and cooperatives to obtain macaúba oil because it is a process that allows for a longer shelf life and longer conservation time (3).

In obtaining macaúba almond oil, an amount of residue is generated that is rich in nutrients, mainly proteins and fibers, therefore, it can be used as an ingredient to compose animal feed and/or human food (1, 2). A viable alternative is the use of the residue for the elaboration of flour for commercialization and/or use in the enrichment of food products, in this aspect, there is a tendency to use flours from various fruits in the elaboration of bakery products, such as breads, biscuits, pasta, among others (3).

Biscuits are widely consumed products, have a long shelf life, are well accepted, especially by children, and are easy to incorporate alternative ingredients. In addition, factors such as ease of manufacture, marketing, and distribution make biscuits of high commercial interest, being obtained by kneading and cooking dough prepared with flour and other ingredients (4). The nutritional quality of biscuits can be improved through the incorporation of bioactive ingredients in their formulation, meeting nutritional needs, and consequently promoting health (3).

Of particular importance, sequilhos-type biscuits have been a vehicle for studies of enrichment of formulations, either for economic, nutritional reasons or also for the flexibility in the characteristics of the final product (5). In this sense, several studies reported new formulations of sequilhos using ingredients such as soybean residue flour (6), pequi pulp flour (7), mixed amaranth and corn flour (8), and cashew peduncle residue flour (9).

In this aspect, the present work aims to elaborate a macaúba almond flour, evaluate its physico-chemical properties and nutritional characteristics, and introduce it in sequilhos-type biscuits formulations. The development of the present biscuits is an alternative proposal to improve the quality of this type of product in addition to the use of macaúba almond residue.

## Materials and methods

### Botanical collection and identification

Fruits of *A. intumescens* (“macaúba”) were collected in an area of Chapada do Araripe (Sítio Arajara), municipality of Barbalha, CE, Brazil. Exsiccate of the species (#9710) is deposited in the Herbarium Caririense Dárdano Andrade Lima (HCDAL) of the Universidade Regional do Cariri (URCA).

### Preparation of the flour

After collection, the fruits were separated, excluding those that had mechanical lesions and/or the presence of microorganisms, then sanitized under running water and immersed for 15 min in a 1% sodium hypochlorite solution. The fruits were peeled and then manually pulped using stainless steel knives. The almond was obtained by partitioning the fruits with the aid of mechanical press (Brand Ribeiro, Model RP0004). Residual cake was obtained from 400 g of macaúba almond after oil extraction in hydraulic pressure (force of 15 t/cm^2^) for 2 h. This mechanism is normally used by industries and cooperatives to obtain macaúba oil.

In the preparation of almond flour, the methodology used was the one proposed by Sousa et al. (10). The residual cake from the extraction was ground in an industrial blender (Brand Skymsen, Model LSB-25 SN 010097) and sieved in a sieve with a mesh size of 0.250 mm (60 mesh). The flour obtained was conditioned for 48 h in plastic packaging and stored at room temperature around 26°C with a relative humidity of 40%.

### Formulation of the sequilhos

Four formulations of sequilhos-type biscuits were elaborated following the methodology proposed by Sousa et al. (7), with adaptations. In the preparation of the sequilhos, the standard formulation (S0) without the addition of macaúba flour was prepared by manually mixing sugar, egg, butter, and corn starch, totaling a mass of 265.20 g. The percentage of flour added in the formulations (S1 2%), (S2 4%), and (S3 6%) was calculated in relation to the mass of the standard formulation (Table 1).

**TABLE 1:** Formulations of the sequilhos-type biscuits under different proportions of macaúba almond flour.

Ingredients	Formulations			
	S0	S1	S2	S3
Regular sugar (g)	38.30	38.30	38.30	38.30
Butter (g)	83.30	83.30	83.30	83.30
Egg/yolk (g)	10.30	10.30	10.30	10.30
Maize starch (g)	133.30	133.30	133.30	133.30
Macaúba almond flour (g)	0.00	5.304	10.608	15.912

S0, standard formula; S1, 2% formula; S2, 4% formula; S3, 6% formula.

The sequilhos were modeled with the aid of a stainless steel knife to obtain standard sizes, in a round shape. Were placed in a greased form with wheat flour and butter and baked in an automatic electric oven (Brand Venâncio, Model CS-4 S) preheated to 180°C for 15–25 min. After 20 min in the oven the sequilhos were removed and cooled for approximately 30 min at room temperature. The sequilhos were stored for 72 h in sealed plastic packages and kept at room temperature around 26°C with a relative humidity of 40%.

### Physico-chemical determinations of flour and sequilhos

The physico-chemical analyses of the flour and sequilhos were carried out in triplicates. Moisture was determined by the desiccation loss method in direct drying in an oven (Brand Memmert, Model 400 S/N B4980457) at 105°C for 24 h (10). Lipids were determined by the Soxhlet method with extraction of the hexane fraction by intermittent flow and carbohydrates obtained by difference (10). Proteins were determined by the Kjeldahl method, where the sample was digested and distilled, using a factor of 6.5 to convert nitrogen into protein (10). The ash by the incineration residue method in a muffle furnace (Brand Solidsteel, Model SSFMr) at 550°C for 6 h (10). The pH was measured in a potentiometer with direct determination and the acidity (% oleic acid equivalent) was determined by the NaOH titration method (10).

### Nutritional information of the sequilhos

The calculations of the total energy value (TEV) of the sequilhos were based on RDC n°*C* 359 (11). The values were expressed referring to a portion of 30 g (12 U) and a diet of 2.000 Kcal/day. The energy value was calculated by adding and multiplying the macronutrients (protein, carbohydrate, and lipid) by the amount of energy supplied by each one (% protein × 4 Kcal + % lipid × 9 Kcal + % carbohydrate × 4 Kcal) expressed in Kcal/100 g.

### Microbiological analysis of the sequilhos

The microbiological analyses carried out on the sequilhos are those indicated for the food group: biscuits and crackers, without filling, with or without topping, including honey bread, biscuits, and the like (12). The microorganisms evaluated were: thermotolerant coliforms at 44°C, molds, and yeasts. All analyses were performed in triplicates, in order to obtain consistent and reliable results.

The presence of thermotolerant coliforms was confirmed by inoculation in 2% bright bile green broth incubated at 44°C. The presence of gas in the inverted Durhan tubes evidences the fermentation of the lactose present in the medium. The reading took place after 24–48 h of incubation using the most probable number (MPN) (13).

The mold and yeast count was performed using the standard plate counting method, in which 25 g of the sample was homogenized in 225 ml of peptone water with consequent serial dilutions. The number of colony forming units per gram (CFU/g) was determined by inoculation in potato dextrose agar acidified with a 10% tartaric acid solution at a temperature of 25 ± 1°C for 5–7 days (13).

### Statistical analysis

The data were expressed in means ± SD. The values were subjected to analysis of variance (ANOVA) by Tukey’s *post hoc* test at the 5% level which was used to separate the means using GraphPad Prism 5.0. The obtained value *p* < 0.05 should be considered significant.

## Results and discussion

### Physicochemical determinations of flour and sequilhos

Table 2 presents the results of the physico-chemical properties of macaúba almond flour and of sequilhos formulations in different proportions of the flour. Moisture represents the water content of the food and the lower the water content, the more stability the food will have during its shelf life. Thus, a reduced value of moisture contributes less to the proliferation of microorganisms and deterioration by physicochemical reactions (14). Almond flour moisture was 4.29%. A similar result was obtained for pequi and babassu almond flour, corresponding to 4.32 and 3.05%, respectively (1).

**TABLE 2:** Nutritional composition of macaúba almond flour and sequilhos-type biscuits per 100 g of product.

Parameters	Flour	Formulations			
		S0	S1	S2	S3
Moisture (%)	4.29 ± 0.13	5.03 ± 0.05^a^	5.05 ± 0.15^a^	7.98 ± 0.70^b^	8.13 ± 0.20^b^
Carbohydrates (%)	44.46 ± 2.13	71.44 ± 1.00^a^	73.37 ± 1.90^a^	70.91 ± 1.50^a^	67.17 ± 1.60^b^
Lipids (%)	28.74 ± 1.51	21.77 ± 0.82^a^	19.59 ± 0.14^a^	18.77 ± 0.80^a^	21.84 ± 0.36^a^
Proteins (%)	20.06 ± 1.10	0.84 ± 0.14^a^	1.16 ± 0.20^b^	1.50 ± 0.08^b^	1.92 ± 0.30^b^
Ashes (%)	2.45 ± 0.19	0.92 ± 0.00^a^	0.83 ± 0.00^a^	0.84 ± 0.15^a^	0.94 ± 0.01^a^
pH	5.33 ± 0.02	5.88 ± 0.05^a^	5.93 ± 0.02^a^	5.71 ± 0.04^a^	5.52 ± 0.04^a^
Titratable acidity (%)*	1.13 ± 0.11	0.11 ± 0.02^a^	0.10 ± 0.00^a^	0.14 ± 0.01^a^	0.13 ± 0.00^a^

S0, standard formula; S1, 2% formula; S2, 4% formula; S3, 6% formula. *% oleic acid equivalent. Results are expressed as mean ± SD. Means followed by the same letter in the same row do not differ statistically by Tukey’s test (p < 0.05).

The moisture values of the sequilhos formulations ranged from 5.03 to 8.13%. Moisture increased in the formulations added to the flour. This fact may be due to the protein and/or fiber content that has the capacity to retain a greater amount of water in the final product (15, 16). An increase in moisture was also observed in cookies with the addition of guavira residue flour, with values ranging from 8.36 to 11.95% (15). The results are also in agreement with the values obtained for cookies with partial replacement of rice flour with soy flour. The increase in moisture content was 8.68% in the standard formulation to 9.30% in the biscuit formulation with 20% soy flour addition (16).

The carbohydrate content in the flour was 44.46%. Overall, flours demonstrate relevant carbohydrate values (17). Pequi and babassu almond flours showed carbohydrate values corresponding to 47.28 and 52.23%, respectively (1). Carbohydrates are the major components in dry matter of plant origin (18). Carbohydrate contents differed significantly between formulations, with values ranging from 67.17 to 73.37%. Sequilhos have shown relevant variations in carbohydrate contents, such as formulations added with concentrations of coffee, ranging from 77.03 to 79.93% (19) and with pequi pulp flour, ranging from 65.42 at 71.40% (7).

A reduction in carbohydrates was observed in the formulations with greater addition of flour. This fact may be related to the increase in lipid and moisture contents in these formulations. Changes in values in one or more of the following parameters in a sample, lipids, proteins, moisture, and ash, typically change the carbohydrate content (4, 20). Cookie formulations with greater addition of babassu pie and cowpea flour also showed a reduction in carbohydrates, with values ranging respectively from 61.85 to 57.84 and 57.56 to 50.17% (20, 21). Cake formulations added with *Hancornia speciosa* (mangaba) pulp flour were also observed to reduce carbohydrates, with values ranging from 51.53 to 45.89% (22).

The lipid content in the flour was 28.74%. The almond, because it is rich in fixed oil, normally generates a flour with a high lipid content (3). However, the oil extraction process collaborated to obtain a flour with lower lipid content. This allows for a longer shelf life of the flour due to a lower susceptibility to lipid oxidation (17). On the other hand, this process promotes a better use of the almond, because, in addition to the oil, the residues obtained from the extraction can be used to generate several products, in this work, it was flour and sequilhos.

The lipid values in the formulations of sequilhos with the addition of flour were statistically equal to the standard formulation. However, there was a tendency to increase this parameter in the formulations with the addition of flour. In sequilhos added with pequi pulp flour, statistical differences were observed between the formulations and the values ranged from 14.72 to 18.54% (7). The addition of mixed amaranth and corn flour in sequilhos formulations promoted an increase in the lipid content between the formulations, whose values ranged from 6.76 to 10.15% (8).

The protein content in the flour corresponded to 20.06%. The result is similar to that obtained for the flour produced from pequi and babassu almonds, with values of 20.50 and 22.38%, respectively (1). The results showed a significant increase in proteins in the formulations added to the flour in relation to the standard formulation, with values ranging from 0.84 to 1.92%. Even though there was no significant difference in the protein content between the formulations with the addition of flour, the values indicate a trend toward an increase in this parameter. In sequilhos with pequi pulp flour, protein contents ranged from 5.37 to 9.18% (7), and with soybean residue flour, they ranged from 0.88 to 5.32% (6). Both studies also showed an increase in protein content between the formulations with the addition of flour in relation to the control formulation.

The ash content found in the flour was 2.45%. The value was relevant and similar to that observed for other flours. It has been reported ash contents ranging from 2.17 to 3.85% in residual cake flour from pequi, macaúba and buriti pulp, and babassu almond flour (1, 17). This parameter is relevant because it can be used as a general measure of the quality of minerals contained in the product, determination of foods rich in minerals and is also a starting point for the analysis of specific minerals (17).

The ash contents in the formulations added to the flour were statistically equal to the control formulation. However, there were values indicating a tendency to increase ash in the formulations with the addition of flour. In the evaluation of sequilhos added with flour from dehydrated cashew peduncle residue, values ranging from 0.81 to 1.67% and a significant increase in ash in the formulations were obtained (9). Ash content was increased in sequilhos added with flours from unconventional parts of pumpkin and watermelon, with values ranging respectively from 0 to 0.51% (23).

The pH and acidity values of the flour were respectively 5.33 and 1.13%. The pH value indicated a low acidity flour, because the foods can be divided into low acidity (pH >4.5), acidic (pH between 4.0 and 4.5), and very acidic (pH <4.5) (17). Acidity and pH are useful in determining the state of conservation of foods, as well as for compliance with legislation and quality control parameters (24). For the sequilhos, the ash contents were statistically equal to the control. In the sequilhos added to pequi pulp flour, the values for acidity ranged from 1.10 to 1.63% and pH from 6.84 to 7.14 to 5.96 (9). In both works, the values of the added formulations were statistically equal to the control.

### Nutritional information of the sequilhos

Table 3 shows the energy values of the sequilhos formulations. It was found 172.50 Kcal/100 g for S0, 142.33 Kcal/100 g for S1, 137.57 Kcal/100 g for S2, and 141.90 Kcal/100 g for S3. The values correspond respectively to 8.62, 7.11, 6.87, and 7.09% of the daily caloric needs of an adult individual. In sequilhos with the addition of unconventional parts of pumpkin and watermelon, the caloric values ranged from 117.60 to 132.90 Kcal/100 g (21), and with the addition of pequi pulp flour, they ranged from 131.26 to 139.88 Kcal/100 g (7).

**TABLE 3:** Nutritional information of sequilhos-type biscuits enriched with different concentrations of macaúba almond flour.

Components	Formulations			
	S0	S1	S2	S3
Energetic value (Kcal/100 g)	172.50	142.33	137.57	141.90
Carbohydrates (g)	21.40	22.00	21.30	20.15
Lipids (g)	9.50	5.90	5.60	6.55
Proteins (g)	0.25	0.35	0.45	0.60

S0, standard formula; S1, 2% formula; S2, 4% formula; S3, 6% formula.

The amounts of carbohydrates and lipids in relation to proteins indicated a more effective contribution of these nutrients to the caloric value of the formulations. In addition, flours are an excellent source of energy, normally given their carbohydrate and lipid content (17). However, the extraction of almond oil made it possible to obtain a flour with a lower lipid content. Thus, the use of flour in the sequilhos formulations allowed to obtain a less caloric product. Flours from pequi and babassu almonds after extraction of the fixed oil showed reduced levels of lipids (1). The babassu almond pie after oil extraction was used in the preparation of cookies and a less caloric product was observed, with values ranging from 143.50 to 152.00 Kcal/100 g (20).

### Microbiological analysis of the sequilhos

Microbiological analyses are extremely important to verify the hygienic-sanitary conditions of food, the risks that they can present to health and how long they can have a shelf life. Through the results of the microbiological analysis, it can be seen that the sequilhos met the standards required by legislation (13) for thermotolerant coliforms at 44°C, molds and yeasts, attesting to their microbiological quality and processing in accordance with good manufacturing practices (Table 4).

**TABLE 4:** Microbiological quality of sequilhos-type biscuits under different concentrations of macaúba almond flour.

Microorganisms	Unit	Formulations				AML
		S0	S1	S2	S3	
Thermotolerant coliforms at 44°C	MPN/g	<3	<3	<3	<3	10^2^
Molds and yeasts	CFU/g	<10	<10	<10	<10	10^4^

S0, standard formula; S1, 2% formula; S2, 4% formula; S3, 6% formula. CFU, colony-forming units; MPN, most probable number; AML, allowed microbiological limit (12).

Food is considered safe when it does not pose a risk to the health of consumers. Food is vulnerable to contamination by various agents, which can trigger various foodborne diseases, which can be derived from pathogenic microorganisms or the toxins produced by them (25). The presence of coliforms suggests poor hygienic conditions of the place, the product, and/or handling, and they can be transmitted by handlers, insects, or water. In relation to molds, some species form so-called mycotoxins that can cause acute intoxication as well as chronic health effects including cancer, damage to genetic material and damage to the immune system, and in turn, yeasts can transmit diseases harmful to health (26).

## Conclusion

Macaúba almond flour was a viable ingredient for the formulation of sequilhos-type biscuits, highlighting the nutritional content. The microbiological analyses highlight adequate sanitary conditions in the preparation of the sequilhos and indicate that they are suitable for human consumption. The sequilhos showed physicochemical changes with a significant increase in protein levels. The use of flour for the nutritional enrichment of this type of biscuit can be put to good use, recommending an increase of mainly 6%.

## Data availability statement

The original contributions presented in the study are included in the article/supplementary material, further inquiries can be directed to the corresponding author.

## Author contributions

ES: conceptualization. MF and LP: methodology. MS: formal analysis and investigation. HC, AR, and ES: project administration. JV, FS, CL, HC, AR, HmA, ZA, and HaA: visualization. AR, JA, HmA, ZA, and HaA: writing—review and editing and funding acquisition. All authors have read and agreed to the final version of the manuscript.
